# Rational design *via* dual-site aliovalent substitution leads to an outstanding IR nonlinear optical material with well-balanced comprehensive properties†

**DOI:** 10.1039/d2sc03760b

**Published:** 2022-09-07

**Authors:** He-Di Yang, Mao-Yin Ran, Sheng-Hua Zhou, Xin-Tao Wu, Hua Lin, Qi-Long Zhu

**Affiliations:** State Key Laboratory of Structural Chemistry, Fujian Institute of Research on the Structure of Matter, Chinese Academy of Sciences Fuzhou Fujian 350002 China linhua@fjirsm.ac.cn qlzhu@fjirsm.ac.cn; College of Chemistry, Fuzhou University Fujian 350002 China; University of the Chinese Academy of Sciences Beijing 100049 China; Fujian Science & Technology Innovation Laboratory for Optoelectronic Information of China Fuzhou 350002 China

## Abstract

The acquisition of a non-centrosymmetric (NCS) structure and achieving a nice trade-off between a large energy gap (*E*_g_ > 3.5 eV) and a strong second-harmonic generation (SHG) response (*d*_eff_ > 1.0 × benchmark AgGaS_2_) are two formidable challenges in the design and development of infrared nonlinear optical (IR-NLO) candidates. In this work, a new quaternary NCS sulfide, SrCdSiS_4_, has been rationally designed using the centrosymmetric SrGa_2_S_4_ as the template *via* a dual-site aliovalent substitution strategy. SrCdSiS_4_ crystallizes in the orthorhombic space group *Ama*2 (no. 40) and features a unique two-dimensional [CdSiS_4_]^2−^ layer constructed from corner- and edge-sharing [CdS_4_] and [SiS_4_] basic building units (BBUs). Remarkably, SrCdSiS_4_ displays superior IR-NLO comprehensive performances, and this is the first report on an alkaline-earth metal-based IR-NLO material that breaks through the incompatibility between a large *E*_g_ (>3.5 eV) and a strong phase-matching *d*_eff_ (>1.0 × AgGaS_2_). In-depth mechanism explorations strongly demonstrate that the synergistic effect of distorted tetrahedral [CdS_4_] and [SiS_4_] BBUs is the main origin of the strong SHG effect and large birefringence. This work not only provides a high-performance IR-NLO candidate, but also offers a feasible chemical design strategy for constructing NCS structures.

## Abstract 2

A new promising IR-NLO material SrCdSiS_4_ with a 2D layered structure has been designed *via* a dual-site isovalent substitution strategy. Remarkably, it is the first report on an alkaline-earth metal-based IR-NLO material that breaks through the wall of *E*_g_ > 3.5 eV and *d*_eff_ > 1 × AgGaS_2_.

## Graphical abstract

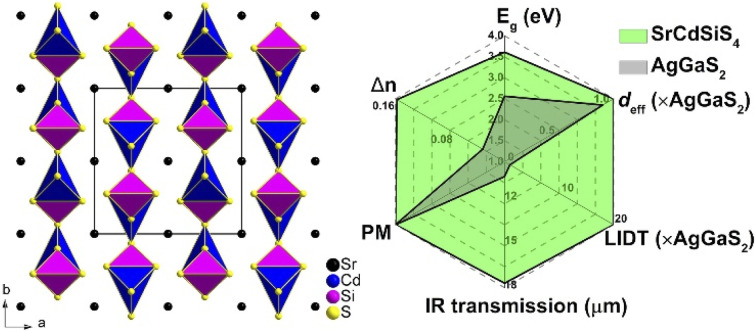

## Introduction

The infrared (IR) laser occupies a more and more important position in military weapons, information storage, precision micromanufacturing and other scientific research.^1–5^ Generally, the IR nonlinear optical (NLO) crystal is an integral part for IR laser generation, which requires a phase-matching (PM) feature, a broad energy gap (*E*_g_), a strong second-harmonic-generation (SHG) intensity (*d*_eff_), a large laser-induced damage threshold (LIDT), an appropriate birefringence (Δ*n*), and a favorable physical and chemical stability.^6^ Chalcopyrite-type materials, AgGaS_2_,^7^ AgGaSe_2_,^8^ and ZnGeP_2_,^9^ are commercially available and exhibit sufficient *d*_eff_ and wide transmittance in the IR region. However, they still suffer from several fatal drawbacks, *e.g.* low LIDT of AgGaS_2_, non-phase-matching (NPM) behavior of AgGaSe_2_ and unexpected multi-phonon absorption of ZnGeP_2_, which hinder their further applications in far-IR regions and high-power lasers. Therefore, it is necessary and urgent to explore new IR-NLO candidates with excellent comprehensive properties.

A non-centrosymmetric (NCS) structure is the prerequisite for a NLO crystal, which is also the first challenge in the design and synthesis of IR-NLO candidates. In order to overcome this problem, various strategies have been developed in the past decades. Among them, chemical substitution is considered to be the simplest and most effective method.^10,11^ On one hand, it can greatly improve the IR-NLO properties of chalcogenides, such as, Li_0.6_Ag_0.4_GaS_2_ (*d*_eff_ = 1.1 × AgGaS_2_) *versus* LiGaS_2_ (*d*_eff_ = 0.4 × AgGaS_2_),^12^ Cu_5_Zn_0.5_P_2_S_8_ (*d*_eff_ = 0.3 × AgGaS_2_) *versus* Cu_3_PS_4_ (*d*_eff_ = 0.03 × AgGaS_2_),^13^ and Sr_1.3_Pb_0.7_GeSe_4_ (*d*_eff_ = 16 × SiO_2_) *versus* Pb_2_GeSe_4_ (*d*_eff_ = 2 × SiO_2_).^14^ On the other hand, it can realize centrosymmetric (CS)-to-NCS structure evolution in the chalcogenide system. Some classic examples include CS La_2_CuInS_5_*versus* NCS La_2_CuSbS_5_,^15^ CS Ba_2_GaAsSe_5_*versus* NCS Ba_2_As_2_Se_5_,^16^ CS BaGa_2_Se_4_*versus* NCS K_0.38_Ba_0.81_Ga_2_Se_4_,^17^ CS K_2_Sb_4_S_7_*versus* NCS K_2_Ag_3_Sb_3_S_7_,^18^ CS Rb_4_Hg_2_Ge_2_S_8_*versus* NCS (Na_3_Rb)Hg_2_Ge_2_S_8_,^19^ CS SrGeO_3_*versus* NCS SrGeOSe_2_,^20^ CS SnBr_2_*versus* NCS Sn_7_Br_10_S_2_,^21^ and CS Rb_4_P_2_S_6_*versus* NCS RbBiP_2_S_6_.^22^ Notably, compared to a large number of structural transformations achieved by single-site substitution mentioned above, examples of dual-site and multi-site substitution are rarely reported.^23,24^

Among the essential conditions for a promising IR-NLO candidate, a large *E*_g_ and a strong *d*_eff_ are not only the most vital factors but also the most challenging to achieve concurrently due to their incompatibility. Metal chalcogenides have been considered as promising candidates for IR-NLO materials, and nearly a thousand novel NLO-active chalcogenides have been discovered in the past few decades.^25–31^ Unfortunately, there are only 6 PM chalcogenides that can meet the preferred requirement for a useful IR-NLO crystal, that is, a nice trade-off between a large *E*_g_ (> 3.5 eV) and strong *d*_eff_ (> 1.0 × benchmark AgGaS_2_), see Table S1 in the ESI for details.† As summarized in Table S1,† some useful information can be obtained as follows: (1) all of them are sulfides; (2) most significant structural features are two-dimensional (2D) or three-dimensional (3D) structures that are constructed from tetrahedral [MS_4_] basic building units (BBUs) (M = metal elements); (3) the filled cations are mainly alkali metals (A) or polycations. Nevertheless, a similar example based on an alkaline-earth metal (AE) as a filled cation is still not reported to date.

Recently, our research focuses on the ternary AE–M^III^–Q system (M^III^ = group IIIA metal Ga, In), hoping to obtain NCS chalcogenides. The tetrahedral [M^III^Q_4_] BBUs are the beneficial NLO-active units for achieving a large *d*_eff_, while the introduction of AE elements into this system may have the additional advantage of enlarging the *E*_g_, which may help to increase the LIDT once an IR-NLO crystal is obtained.^32–34^ Our systematic exploratory efforts have led to the discovery of a known ternary sulfide in this family, namely, SrGa_2_S_4_.^35^ It exhibits a unique 2D [Ga_2_S_4_]^2−^ layer that is constructed from common NLO-active [GaS_4_] units and possesses a wide optical *E*_g_ (3.93 eV) and a large theoretical birefringence (Δ*n* = 0.147@2050 nm). Unfortunately, the CS space group of *Fddd* (no. 70) makes this sulfide NLO inert, that is, it does not display any SHG signal under laser irradiation. Inspired by the aforementioned chemical substitution strategy and detailed structural analysis, we are eager to realize the CS-to-NCS structural evolution *via* the replacement of two Ga^III^ sites by “M^I^ + M^V^” or “M^II^ + M^IV^” in such a 2D layer. We term this the “dual-site aliovalent substitution” strategy.

Guided by a dual-site aliovalent substitution strategy, a novel quaternary NCS sulfide SrCdSiS_4_ was successfully discovered herein. Remarkably, SrCdSiS_4_ exhibits the PM feature and excellent IR-NLO performances, including a strong *d*_eff_ (1.1 × AgGaS_2_), wide *E*_g_ (3.61 eV), ultra-high LIDT (20.4 × AgGaS_2_), broad transmission range (0.33–18.19 μm) and suitable Δ*n* (0.158@2050 nm), which indicates that it is a promising candidate for IR-NLO materials and eliminates the disadvantageous factors of commercial chalcopyrite-type chalcogenides. Moreover, SrCdSiS_4_ is also the first example of an alkaline-earth metal-based IR-NLO material that breaks through the incompatibility between a large *E*_g_ (>3.5 eV) and a strong PM *d*_eff_ (>1.0 × AgGaS_2_). In this work, a systematic study of the syntheses, structural evolution, NLO and linear optical properties, and the in-depth mechanism is reported as well.

## Results and discussion

**Fig. 1:**
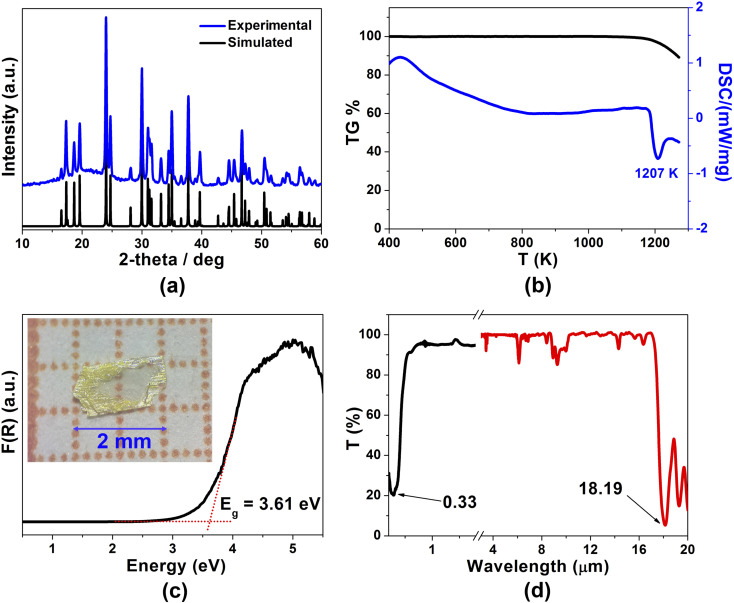
Experimental characterization results of SrCdSiS_4_: (a) experimental (blue) and simulated (black) powder XRD curves; (b) TG and DSC diagrams; (c) solid-state UV-vis-NIR diffuse reflectance spectrum (inset: photograph of a polished crystal); (d) optical transmittance spectra from UV-vis to IR region.

In this study, light-yellow crystals of SrCdSiS_4_ were prepared by a high-temperature solid-state reaction between stoichiometric SrS, CdS, Si, and S at 1123 K using CsI as the flux. The purity of the polycrystalline sample was checked by powder X-ray diffraction (XRD) analysis (Fig. 1a), and energy-dispersive X-ray spectroscopy (EDX) provides average atomic ratios of 1.09/1.10/1/4.13 for Sr, Cd, Si, and S elements (Fig. S1†), which are close to theoretical values determined from single-crystal XRD results. As shown in Fig. 1b, SrCdSiS_4_ exhibits desirable thermal stability below 1207 K under N_2_ condition and decomposes to Sr_2_SiS_4_ and CdS at higher temperatures (Fig. S2†). The UV-vis and near-IR absorption spectra of SrCdSiS_4_ reveal an optical *E*_g_ of 3.61 eV (see Fig. 1c) based on the Kubelka Munk function,^36^ which is not only keeping the advantage of the wide *E*_g_ of the parent compound SrGa_2_S_4_ (3.93 eV, as plotted in Fig. S3†) but is also considerably wider than those of commercial IR-NLO materials AgGaS_2_ (2.56 eV),^37^ AgGaSe_2_ (1.83 eV)^38^ and ZnGeP_2_ (2.0 eV).^39^ Notably, such an ultra-wide *E*_g_ of SrCdSiS_4_ can effectively avoid two- or multi-photon absorptions under the incident normal laser, which is helpful to obtain a high LIDT. In addition, the transmittance spectrum (Fig. 1d) recorded from a well-polished single crystal piece indicates that SrCdSiS_4_ exhibits a wide transparent window from 0.33 μm (UV-vis region) to 18.19 μm (far-IR region), which can cover two notable atmospheric windows (3–5 μm and 8–12 μm). Remarkably, such a transparent range is wider than those of distinguished IR-NLO materials AgGaS_2_ (0.48–11.4 μm),^39^ ZnGeP_2_ (0.74–12 μm),^39^ AgGaSe_2_ (0.76–17 μm)^39^ and other recently reported IR-NLO candidates.^40–45^

**Fig. 2:**
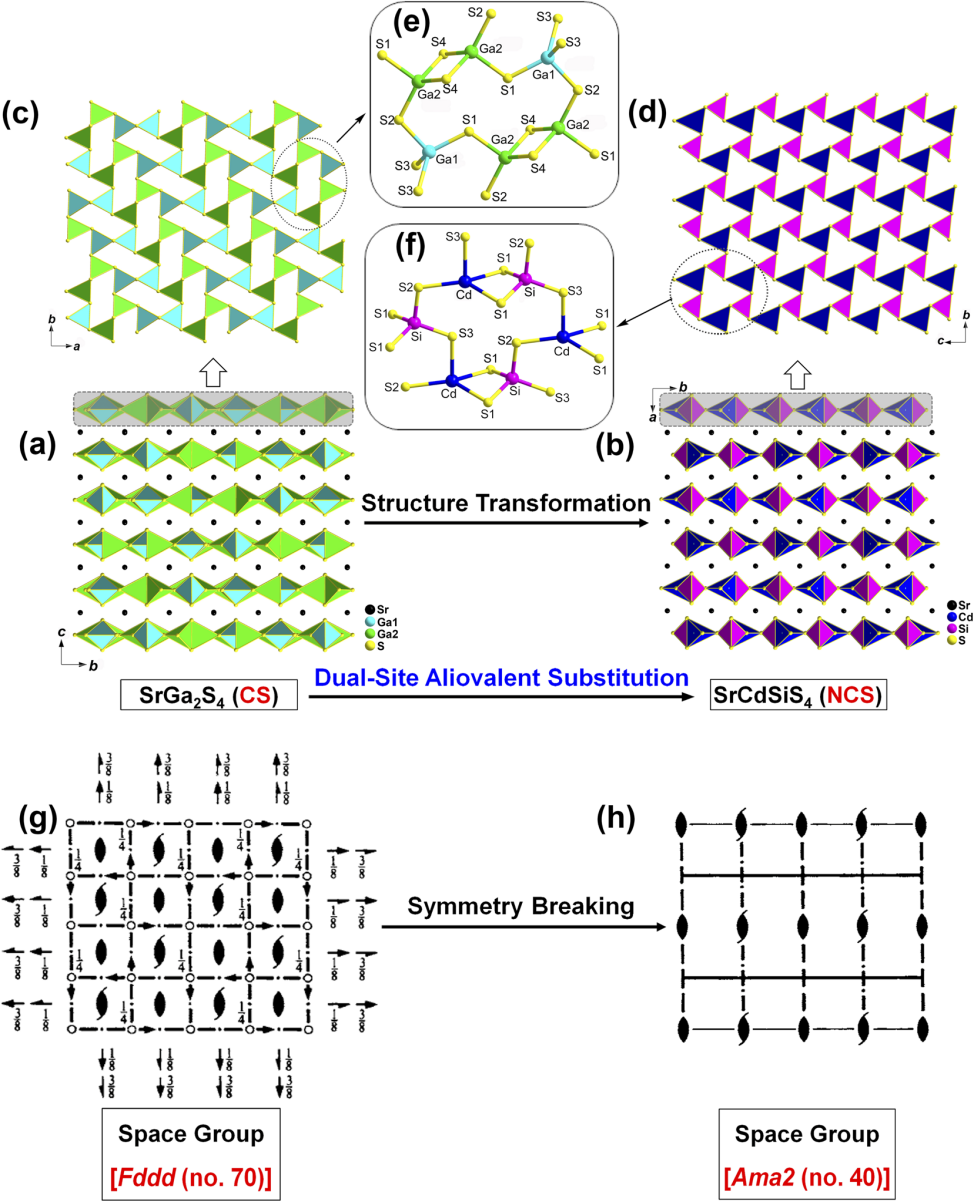
Structural evolution from CS SrGa_2_S_4_ to NCS SrCdSiS_4_: (a and b) view of the crystal structure of SrGa_2_S_4_ and SrCdSiS_4_ along the *bc* and *ab* planes, respectively; (c and d) 2D tetrahedral-stacking [Ga(1)Ga(2)S_4_]^2−^ and [CdSiS_4_]^2−^ layers *via* edge- and corner-sharing with the repeat 12-member rings (dashed part) marked viewed along the *ab* and *bc* planes, respectively; (e and f) the ball-and-stick models of the 12-member-ring [Ga(1)_2_Ga(2)_4_S_16_] and [Cd_3_Si_3_S_16_] functional primitives with the atom number marked; (g and h) spatial symmetry operation change from CS [high symmetry *Fddd* (no. 70)] to NCS [low symmetry *Ama*2 (no. 40)].

The structural evolution from CS SrGa_2_S_4_ to NCS SrCdSiS_4_ based on the dual-site aliovalent substitution strategy is illustrated in Fig. 2. Comparison of their structures shows that they belong to the same orthorhombic system and possess tetrahedral [MS_4_] BBUs in their 2D layered structures. However, they still have several significantly different characteristics in their structures: (i) SrCdSiS_4_ crystallizes in the space group of *Ama*2 (no. 40), while SrGa_2_S_4_ adopts the space group of *Fddd* (no. 70), see Table 1 for details; (ii) the asymmetric unit of SrCdSiS_4_ has 6 crystallographically independent sites (*i.e.*, 1 Sr, 1 Cd, 1 Si, and 3 S atoms) and the *Z* value (number of molecules in a unit cell) is 4, which are different from those of SrGa_2_S_4_ [9 unique sites, namely, 3 Sr, 2 Ga, and 4 S atoms) and *Z* = 32], see Tables 1 and 2 for details; (iii) note that the repeated functional primitive, namely the 12-member-ring [Cd_3_Si_3_S_16_] (including 3 [CdS_4_] and 3 [SiS_4_] BBUs, see the dashed part in Fig. 2f) exists in each 2D [CdSiS_4_]^2−^ layer of SrCdSiS_4_ (Fig. 2d), but in SrGa_2_S_4_, the repeated 12-member-ring [Ga_6_S_16_] in each 2D [Ga_2_S_4_]^2−^ layer consists of 2 [Ga(1)S_4_] and 4 [Ga(2)S_4_] BBUs (see the dashed part in Fig. 2c and e); (iv) the [SrS_8_] polyhedra are more highly distorted in SrCdSiS_4_ than those in SrGa_2_S_4_, *e.g.*, the larger difference (Δ*d*) between the Sr–S bonds (Δ*d* (Sr–S) = 0.15 Å) in SrCdSiS_4_ than that (0.03 Å) in SrGa_2_S_4_, and a similar trend also occurred in the tetrahedral [MS_4_] BBUs, see Fig. S4 and Tables S2 and S3 for details.† In a word, the dual-site aliovalent substitution led to the above-mentioned obvious changes in their crystal structures, thus realizing the CS-to-NCS structural transformation from ternary SrGa_2_S_4_ to quaternary SrCdSiS_4_. Moreover, the detailed symmetric operation change shown in Fig. 2g and h clearly displays the evolution of symmetry breaking, that is, the loss of the different glide planes and the inversion centre from CS SrGa_2_S_4_ [high symmetry *Fddd* (no. 70)] to NCS SrCdSiS_4_ [low symmetry *Ama*2 (no. 40)].

**Table 1:** Crystallographic data and refinement details for SrCdSiS_4_ and SrGa_2_S_4_

Empirical formula	SrCdSiS_4_	SrGa_2_S_4_
Formula weight	356.35	355.30
Temperature (K)	293(2)	293(2)
Crystal system	Orthorhombic	Orthorhombic
Space group	*Ama*2 (no. 40)	*Fddd* (no. 70)
*a* (Å)	10.2821(7)	12.2216(5)
*b* (Å)	10.1551(9)	20.5008(9)
*c* (Å)	6.3699(5)	20.8426(10)
*V* (Å^3^)	665.12(9)	5222.2(4)
*Z*	4	32
*D*_c_ (g cm^−3^)	3.559	3.615
*μ* (mm^−1^)	12.520	17.481
GOOF on *F*^2^	1.204	1.174
*R*_1_, w*R*_2_ (*I* > 2*σ* (*I*)) a	0.0212, 0.0533	0.0228, 0.0591
*R*_1_, w*R*_2_ (all data)	0.0217, 0.0594	0.0338, 0.0627
Largest diff. peak and hole (e Å^−3^)	0.81, −0.49	0.69, −1.03
Flack parameter	0.007(12)	

a *R*_1_ = Σ‖*F*_o_| − |*F*_c_‖/Σ|*F*_o_|, w*R*_2_ = [Σ*w*(*F*_o_^2^ − *F*_c_^2^)^2^/Σ*w*(*F*_o_^2^)^2^]^1/2^

**Table 2:** Atomic coordinates and equivalent isotropic displacement parameters (Å^2^) of SrCdSiS_4_ and SrGa_2_S_4_

Atom	*Wyckff*	*X*	*y*	*Z*	*U*_eq_(Å)^a^
**SrCdSiS**_**4**_					
Sr	4*a*	0	0	0	0.0165(4)
Cd	4*b*	0.75	0.83086(9)	0.4430(2)	0.0251(4)
Si	4*b*	0.75	0.7178(3)	0.9879(5)	0.0124(7)
S1	8*c*	0.4166(2)	0.7754(2)	0.6887(3)	0.0165(5)
S2	4*b*	0.75	0.8972(3)	0.8119(4)	0.0150(7)
S3	4*b*	0.75	0.5662(2)	0.7620(5)	0.0171(7)

**SrGa**_**2**_ **S**_**4**_					
Sr1	16*g*	0.875	0.375	0.62685(2)	0.01154(9)
Sr2	8*b*	0.625	0.625	0.625	0.0111(2)
Sr3	8*a*	0.375	0.375	0.875	0.01104(2)
Ga1	32*h*	0.37362(2)	0.51258(2)	0.74942(2)	0.00896(6)
Ga2	32*h*	0.66403(2)	0.44634(2)	0.74984(2)	0.00917(6)
S1	32*h*	0.49464(4)	0.59489(3)	0.74888(4)	0.01004(9)
S2	32*h*	0.48491(4)	0.42197(2)	0.74897(3)	0.00918(9)
S3	32*h*	0.25159(6)	0.50054(3)	0.83226(3)	0.0099(9)
S4	32*h*	0.75071(7)	0.49983(3)	0.66738(3)	0.00993(9)

a *U*_eq_ is defined as one third of the trace of the orthogonalized *U*_ij_ tensor.

**Fig. 3:**
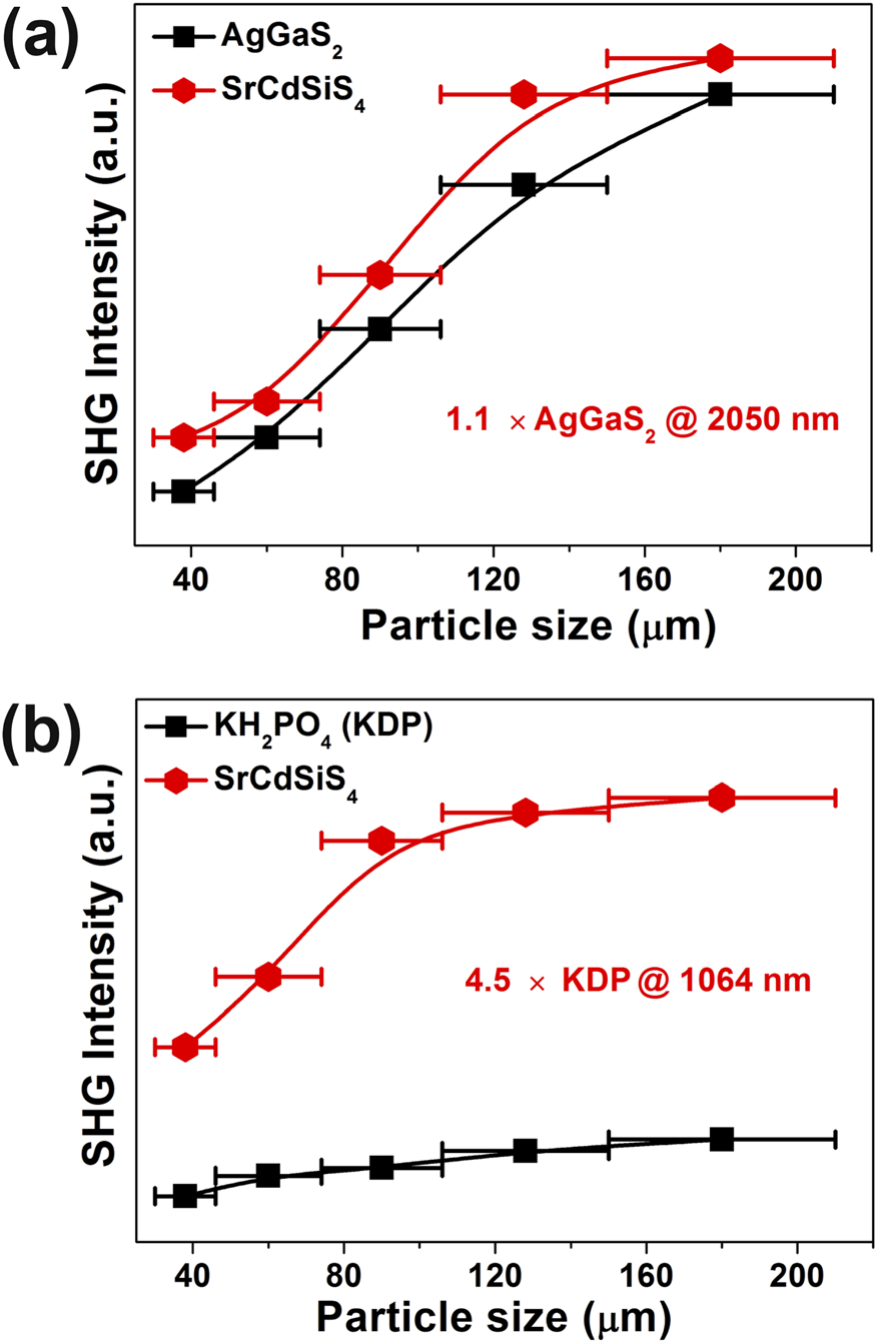
Phase-matching curves for SrCdSiS_4_ and inserted values are the SHG intensities in the particle size range of 150–210 μm: (a) AgGaS_2_ as the benchmark under 2050 nm radiation; (b) KDP as the reference under 1064 nm radiation. The solid curves are drawn as a guide to the eye.

**Fig. 4:**
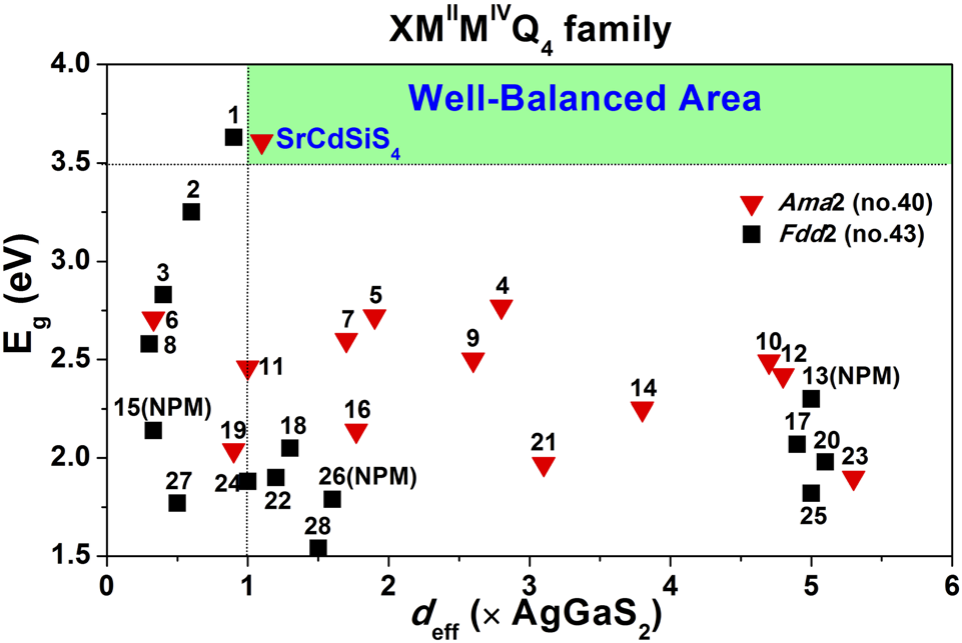
Comparison of *d*_eff_ and *E*_g_ of the XM^II^M^IV^Q_4_ system (X = Eu, Sr, Ba; M^II^ = Mn, Zn, Cd, and Hg; M^IV^ = group-14 elements; and Q = chalcogen) and the green shaded region represents the well-balanced (*E*_g_ > 3.5 eV and *d*_eff_ > 1.0 × AgGaS_2_) area for IR-NLO materials. Details are listed in Table S4.†

Owing to SrCdSiS_4_ possessing the NCS polar structure, we exhaustively investigated and analyzed the NLO performance. Size-dependent SHG effect measurements were performed by using the Kurtz-Perry method^46^ at five different particle size ranges. As illustrated in Fig. 3a, the SHG intensity strength increases with the increase of particle size, indicating that SrCdSiS_4_ can achieve type-I PM in the IR region. Under the same particle size of 150–210 μm, the *d*_eff_ is around 1.1 times that of AgGaS_2_ under a 2050 nm Q-switched laser. We also measured the SHG signals under a 1064 nm laser due to the shorter UV absorption edge of SrCdSiS_4_ (*ca.* 330 nm), giving it the potential to be applied in the UV-vis range. As indicated in Fig. 3b, SrCdSiS_4_ shows a large SHG effect of 4.5 × KH_2_PO_4_ (KDP) with type-I PM nature. Therefore, SrCdSiS_4_ is an excellent dual-band NLO candidate that can be used in both the IR and UV-vis regions. Apart from an adequate SHG response, a large LIDT is also vitally important for an IR-NLO material. So, its LIDT was measured by a single-pulse power technology.^47^ As shown in Fig. S5,† the experimental LIDT of SrCdSiS_4_ of 57.14 MW cm^−2^ in the particle size range of 150–210 μm is around 20.4 times higher than that of benchmark AgGaS_2_ (2.8 MW cm^−2^) under the same condition (1064 nm, 1 Hz, 10 ns). Such a value shows the outstanding laser tolerance of SrCdSiS_4_, indicating its potential in high-power laser applications. As a new member of the XM^II^M^IV^Q_4_ (X = Eu, Sr, Ba; M^II^ = Mn, Zn, Cd, and Hg; M^IV^ = group-14 elements; and Q = chalcogen) system,^24,48–63^ it is necessary to make a detailed comparison with other compounds. A summary of the two key performance parameters (*i.e.*, *d*_eff_ and *E*_g_) of the XM^II^M^IV^Q_4_ family is provided in Fig. 4 and details are listed in Table S4.† Remarkably, SrCdSiS_4_ displays superior IR-NLO comprehensive performances, and this is the first report on an alkaline-earth metal-based IR-NLO material that breaks through the incompatibility between a large *E*_g_ (>3.5 eV) and a strong phase-matching *d*_eff_ (>1.0 × AgGaS_2_) in this system. Furthermore, a more comparative study with other state-of-the-art IR-NLO candidates is worthwhile.^32,64–67^ As shown in Fig. S6 and Table S1,† there are 7 PM IR-NLO chalcogenides with *E*_g_ > 3.5 eV and *d*_eff_ > 1.0 × AgGaS_2_ (Fig. S6a†), which have been selected on the basis of literature research. From the perspective of structural dimension, they are mainly constructed in 3D framework (43%) and 2D layered (43%) structures, and only K_2_BaP_2_S_6_^67^ possess a zero-dimensional (0D) cluster structure (14%) (Fig. S6b†). In addition, they can be divided into four categories according to the kind of filled cation: polycation-based (43%), alkali-metal-based (29%), mixed-cation-based (14%) and alkaline-earth-metal-based (14%) (Fig. S6c†). Note that the central atoms in most of the BBUs are main group elements [*e.g.*, Ga (20%), P (20%), Si (13%), Li (13%) and Ge (7%)] and transition metal elements [*e.g.*, Zn (20%), and Cd (7%)] (Fig. S6d†). The production of SrCdSiS_4_ not only enlarges the proportion of Cd and Si acting as favorable framework cations but also represents the first report of an alkaline-earth metal-based IR-NLO material that breaks through the wall of *E*_g_ > 3.5 eV and *d*_eff_ > 1 × AgGaS_2_.

**Fig. 5:**
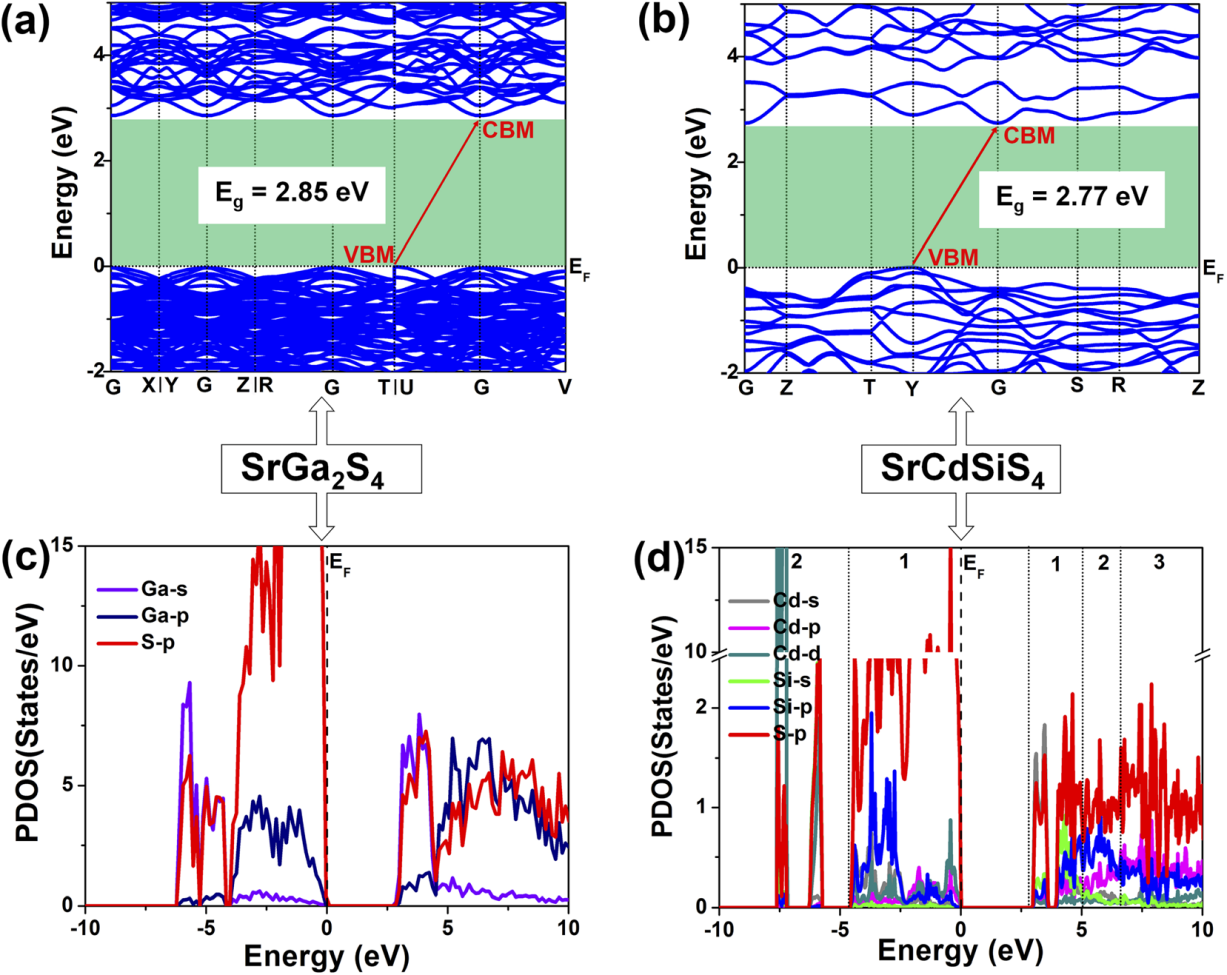
Changes in (a and b) electronic band structures and (c and d) PDOSs (states with less contributions are omitted for better view) from SrGa_2_S_4_ to SrCdSiS_4_ caused by dual-site aliovalent substitution.

**Fig. 6:**
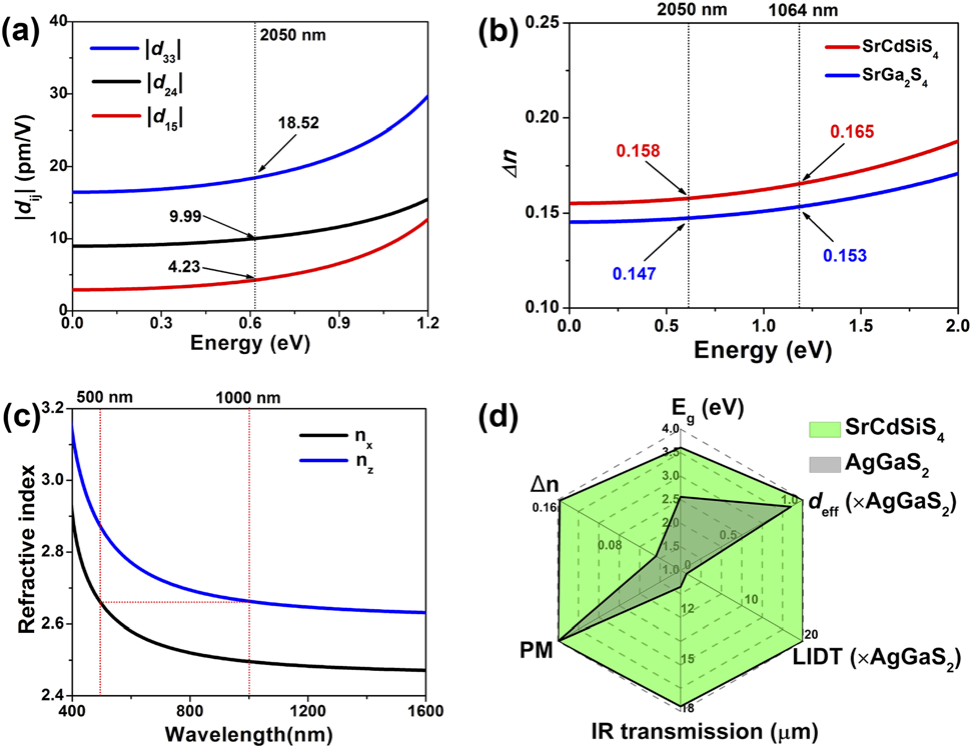
(a) Frequency-dependent SHG coefficients of SrCdSiS_4_; (b) curves of the calculated Δ*n* as a function of energy (eV) for SrGa_2_S_4_ and SrCdSiS_4_; (c) calculated refractive index dispersion curves with the shortest PM cut-off edge at 500 nm; (d) radar chart with six directions (representing *E*_g_, *d*_eff_, LIDT, IR transmission, PM, Δ*n*) to characterize comprehensive IR-NLO performance of SrCdSiS_4_.

Theoretical computations were adopted to better understand the structure–activity relationships of the title compound. According to the electronic structures, the valence band minimum (VBM) and the conduction band maximum (CBM) are at different *k*-points for SrGa_2_S_4_ (Fig. 5a) and SrCdSiS_4_ (Fig. 5b), which indicates that they are indirect *E*_g_ semiconductors. Theoretical results exhibit that the calculated *E*_g_ values are 2.85 eV for SrGa_2_S_4_ and 2.77 eV for SrCdSiS_4_. Such values are smaller than the experimental ones (3.93 eV for SrGa_2_S_4_ and 3.61 eV for SrCdSiS_4_, respectively), which is mainly due to the discontinuity of the exchange correlation energy of the GGA functional.^68–70^ In addition, their partial density of states (PDOSs) in the energy field from −10 to 10 eV are shown in Fig. 5c and d. From the PDOSs, it is found that the contribution in the VBM is mainly from Ga-4s, S-3p orbitals for SrGa_2_S_4_ and S-3p, Si-3p orbitals for SrCdSiS_4_, while the CBM consists of Ga-4p, S-3p orbitals for SrGa_2_S_4_ and Cd-5s, S-3p orbitals for SrCdSiS_4_. The main source of contribution transformed from Ga-4s to Si-3p for the VB and Ga-4p to Cd-5s for the CB after dual-site aliovalent substitution. Accordingly, diverse orbital states finally account for the little difference in optical *E*_g_ and the electron transfer mainly depends on [GaS_4_] (for SrGa_2_S_4_) converting to [CdS_4_] and [SiS_4_] (for SrCdSiS_4_). Moreover, the origin of the SHG response and birefringence (Δ*n*) as the two important NLO indexes were also analyzed in detail. As seen from Fig. 6a, SrCdSiS_4_ has three nonzero independent second-order susceptibility tensors based on the rule of Kleinman's symmetry,^71^ namely, *d*_33_, *d*_24_ and *d*_15_. The calculated values at 2050 nm are *d*_33_ = 18.52, *d*_24_ = 9.99, and *d*_15_ = 4.23 pm V^−1^, respectively. In general, a larger *E*_g_ is usually accompanied by a smaller *d*_eff_, but the largest one is 1.4 times that of reference AgGaS_2_ (*d*_14_ = 13.6 pm V^−1^ at 2050 nm), which is basically consistent with experimental results (about 1.1 times that of AgGaS_2_). In addition, the theoretical static Δ*n* values for SrCdSiS_4_ are 0.158@2050 nm and 0.165@1064 nm, which are higher than those of SrGa_2_S_4_ (Δ*n* = 0.147@2050 nm and 0.153@1064 nm) and sufficiently large to ensure PM features in both UV-vis and IR regions (Fig. 6b). Typically, a significant anisotropic structure is beneficial to produce a large Δ*n*, that is, dual-site aliovalent substitution induces greater structural distortion from SrGa_2_S_4_ to SrCdSiS_4_. Meanwhile, these calculated values are larger than those of commercialized NLO materials, such as AgGaS_2_ (Δ*n* = 0.039@2050 nm),^72^ ZnGeP_2_ (Δ*n* = 0.04@2050 nm)^72^ and KDP (Δ*n* = 0.034@1064 nm).^20^ Besides, the frequency-dependent refractive index diagrams mean that under the premise of PM determined at 2050 nm, the lower limit of the SHG output wavelength is 500 nm (Fig. 6c). Based on theoretical studies and experimental observations, we compared SrCdSiS_4_ with the illustrious IR-NLO crystal AgGaS_2_. As illustrated in the radar chart (Fig. 6d), the green colored shadow is larger than the gray indicating the superior performance of SrCdSiS_4_, including the PM feature, large *E*_g_ (*ca.* 3.61 eV), strong *d*_eff_ (*ca.* 1.1 × AgGaS_2_ at 2050 nm), giant LIDT (20.4 × AgGaS_2_), beneficial Δ*n* (0.158@2050 nm) and broad transparent region (0.33–18.19 μm).

**Fig. 7:**
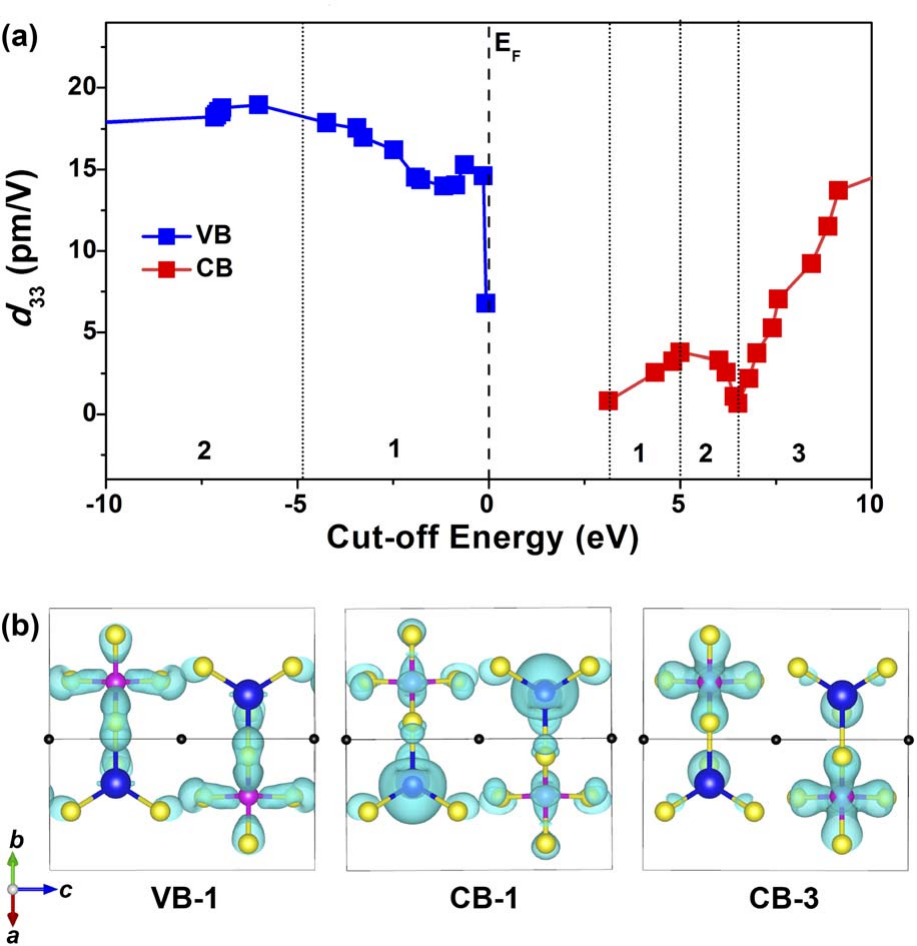
Theoretical analysis of the SHG source for SrCdSiS_4_: (a) variation of cut-off energy (eV) *versus* the largest static *d*_33_; (b) charge–density-maps in the selections (VB-1, CB-1, CB-3) of major contribution. Black atoms: Sr; blue atoms: Cd; pink atoms: Si; yellow atoms: S.

Furthermore, the cut-off energy dependences of the largest static *d*_33_ were analyzed based on a length-gauge formalism method^73,74^ with the purpose of revealing the intrinsic source of the SHG response. As shown in Fig. 7a, *d*_33_ values are trending upward in the range of VB-1 (dominated by the S-3p and Si-3p states, CB-1 (dominated by the S-3p and Cd-5s states) and CB-3 (dominated by the S-3p and Si-3p states). Distinctly, these three regions have a predominant impact on the overall NLO response. Considering the PDOS (Fig. 5d) and the relevant partial charge density profiles (Fig. 7b), the splendid SHG response comes from the collaborative effect of NLO-active [CdS_4_] and [SiS_4_] BBUs, *i.e.*, the 2D [CdSiS_4_]^2−^ alternating arrangement layer.

## Conclusions

In conclusion, employing the ternary CS SrGa_2_S_4_ as the parent structure, a new NCS quaternary SrCdSiS_4_ was successfully designed and synthesized *via* a dual-site aliovalent substitution strategy, whose 2D layered structure consisted of alternately connected [CdS_4_] and [SiS_4_] BBUs through corner- and edge-sharing S atoms. Detailed performance analyses indicated that SrCdSiS_4_ could be a promising candidate for the UV-vis and IR-NLO crystal due to its advantages including a strong SHG intensity (*d*_eff_ = 4.5 × KDP at 1064 nm, or 1.1 × AgGaS_2_ at 2050 nm) with PM feature, a suitable birefringence (Δ*n*_(cal.)_ = 0.165 at 1064 nm, or 0.158 at 2050 nm), a wide transmission window (0.33–18.19 μm), a large *E*_g_ (3.61 eV), and an ultra-high LIDT (20.4 × AgGaS_2_). In addition, theoretical calculations reveal that the large Δ*n* and strong *d*_eff_ are mainly contributed by the tetrahedral [CdS_4_] and [SiS_4_] NLO-active motifs that are nicely arranged in a most favorable stacking. Hopefully, such a simple and effective chemical design strategy can accelerate the discovery of novel NCS materials with advanced NLO properties.

## Data availability

Supporting data for this article is presented in the ESI.†

## Author contributions

Synthesis, characterization and original manuscript: H. D. Yang and M. Y. Ran; theoretical calculations: S. H. Zhou; experimental conception, supervision and manuscript editing: X. T. Wu, H. Lin and Q. L. Zhu. H. D. Yang and M. Y. Ran contributed equally to this work. All authors provided comments and approved the final version of the manuscript.

## Conflicts of interest

There are no conflicts to declare.

## Supplementary Material

SC-013-D2SC03760B-s001

SC-013-D2SC03760B-s002
