# Supplementary material for: Rational design via dual-site aliovalent substitution leads to an outstanding IR nonlinear optical material with well-balanced comprehensive properties
Source: Chem Sci. 2022 Sep 7;13(36):10725–33. doi: 10.1039/d2sc03760b (PMC9491097; doi:10.1039/d2sc03760b)
Supplement: SC-013-D2SC03760B-s001 [file SC-013-D2SC03760B-s001.pdf]

Electronic Supplementary Material (ESI) for Chemical Science.
This journal is © The Royal Society of Chemistry 2022

Electronic Supplementary Information (ESI)

Rational Design via Dual-Site Aliovalent Substitution Leads to an Outstanding IR Nonlinear Optical Material with Well-Balanced Comprehensive Properties

He-Di Yang,‡[a,b] Mao-Yin Ran,‡[a,c] Sheng-Hua Zhou,[a,c] Xin-Tao Wu,[a,d] Hua Lin,*[a,d] and Qi-Long Zhu*[a,d]

[a]*State Key Laboratory of Structural Chemistry, Fujian Institute of Research on the Structure of Matter, Chinese Academy of Sciences, Fuzhou 350002, China*

[b] *College of Chemistry, Fuzhou University, Fujian 350002, China*

[c]*University of the Chinese Academy of Sciences, Beijing 100049, China*

[d]*Fujian Science & Technology Innovation Laboratory for Optoelectronic Information of China, Fuzhou 350002, China*

‡H.-D.Y. and M.-Y.R. contributed equally to this work.

*E-mail: linhua@fjirsm.ac.cn and qlzhu@fjirsm.ac.cn

Electronic Supplementary Information Index

1. Experimental Section

1.1 Materials and Instruments

1.2 Synthesis

1.3 Second-Harmonic Generation (SHG) Measurements

1.4 Laser Induced Damage Threshold (LIDT) Measurements

2. Computational Details

3. Figures and Tables

Figure S1. The characterizations of $SrCdSiS_4$: (a) SEM image and corresponding elemental mapping analysis and (b) EDX results.

Figure S2. The experimental (red) and simulated (black) PXRD of $SrCdSiS_4$ calcining

in 1223 K.

Figure S3. Property measurements for $SrGa_2S_4$: (a) the experimental (red) and simulated (black) PXRD; (b) solid-state diffuse reflectance spectrum.

Figure S4. The coordination environment and bond lengths (Å) of crystallographic independent Sr atoms in (a) $SrCdSiS_4$ and (b–d) $SrGa_2S_4$.

Figure S5. Comparison of the relative SHG and LIDT of $SrCdSiS_4$ and $AgGaS_2$ under the particle size of 150–210 μm.

Figure S6. (a) Comparison of the reported PM IR-NLO chalcogenides with wide energy gap (E_g > 3.5 eV) and strong SHG response (d_{eff} > 1.0 × $AgGaS_2$). Distribution of 7 IR-NLO materials according to: (b) dimension, (c) type of filled cations, (d) central atom of BBUs.

Table S1 Property comparison of the known PM IR-NLO chalcogenides with wide band gaps (E_g > 3.5 eV) and strong SHG responses (d_{eff} > 1.0 × $AgGaS_2$).

Table S2 Selected bond lengths (Å) and angles (deg) of $SrCdSiS_4$.

Table S3 Selected bond lengths (Å) and angles (deg) of $SrGa_2S_4$.

Table S4 Properties comparison of the reported $XM^{II}M^{IV}Q_4$ family (X = Eu, Sr, Ba; M^{II} = Mn, Zn, Cd, and Hg; M^{IV} = group-14 elements; and Q = chalcogen). The sequence of compounds is consistent with Figure 4.

4. References

1. Experimental Section

1.1 Materials and Instruments

All starting reagents were purchased from commercial sources without further purification and handled inside an Ar-filled glovebox. Semi-quantitative energy dispersive X-ray (EDX, Oxford INCA) analyses were performed with an EDX-equipped field emission scanning electron microscope (FESEM, JSM6700F). Powder X-ray diffraction (PXRD) spectrogram were mearsured in a Rigaku Mini-Flex 600 powder diffractometer (Cu-K_{α}, λ = 1.5418 Å). Optical diffuse reflectance spectrum were collected in the region of 200−2500 nm using a Perkin-Elmer Lambda 950 UV-vis-NIR spectrometer at room temperature, the spectrum of the $BaSO_4$ was recorded as baseline. Finally, the instrument will use Kubelka-Munk function to convert diffuse reflectance data into absorbance.[1] Crystals of $SrCdSiS_4$ were measured to obtain IR transmittance on a PerkinElmer Spectrum One FT-IR Spectrometer (400−4000 cm^{-1}). The thermal stability analysis was studied using the NETZSCH STA 449C simultaneous analyser in the atmosphere of nitrogen, the highest temperature was setted at 1273 K.

1.2 Synthesis

The pale-yellow crystals of $SrCdSiS_4$ were obtained by flux method. SrS (3N, Alfa-Aesar), CdS (4N, Aladdin), Si (6N, Alfa-Aesar), S (3N, Aladdin) were weighed in the mole ratio and CsI (5N, Aladdin) as flux. These raw materials (ca. 600 mg) were loaded into quartz silica tubes and subsequently evacuated to 10–3 Pa and sealed, which was heated to 673 K and kept 10 h, then cooled down to 623 K within 300 h after 120 h of insulation at 1123 K, and finally the furnace was shut off. The single crystals were obtained after washing with hot distilled water and ethanol. Besides, they are insensitive to water and air. The colorless $SrGa_2S_4$ materials were synthesized

by high-temperature solid-phase method. Total mass 300 mg of SrS (3N, Alfa-Aesar), Ga (4N, Aladdin) and $SrCl_2$ (2.5N, Alfa-Aesar), S (3N, Aladdin) in a ratio of 1/4/1/7 were weighed and mixed. Then reacted at 1123 K for 50h, other process are the same as $SrCdSiS_4$.

1.3 Single-Crystal Structure determination

The crystal structure of $SrCdSiS_4$ and $SrGa_2S_4$ were determined by single-crystal X-ray diffraction (XRD) on a Saturn 724 diffractometer using graphite-monochromated Mo-K_α radiation (λ = 0.71073 Å) at 293 K and absorption correction were accomplished by multi-scan method. Direct methods[2] and fill-matrix least-squares methods[3] on F^2 based on *SHELX−2014* were taken for solving and refining crystal structure, respectively. Besides, all atoms were refined with anisotropic thermal parameters. Crystal data and structure refinement parameters were given in Table 1. The final refined atomic positions and isotropic thermal parameters are listed in Table 2. Interatomic distances (Å) and bond angles (deg) are displayed in Table S2 and S3. CIFs of $SrCdSiS_4$ and $SrGa_2S_4$ have been submitted with CCDC numbers 2167628 and 2168083, respectively.

1.4 Second-Harmonic Generation (SHG) Measurements

The powder SHG property test was investigated by Kurtz-Perry method[4] using a Q-switched laser radiation. The laser radiation at 2050 nm, 1064 nm were selected as the laser sources and $AgGaS_2$, KH_2PO_4 (KDP) were measured as the benchmark, respectively. Samples of $SrCdSiS_4$, $AgGaS_2$ and KDP were ground and sieved into different granule sizes (30−46, 46−74, 74−106, 106−150, and 150−210 μm) for the phase matching measurements. The frequency-doubled output signals were detected via photomultiplier tube and oscilloscope.

1.5 Laser Induced Damage Threshold (LIDT) Measurements

Crystals of $SrCdSiS_4$ at the scope of 150−210 μm were picked out for LIDT measurement by single pulse measurement method[5] and the similar scope of $AgGaS_2$ single crystal used as reference. The prepared samples were packed into plastic holders with 1 mm in thickness and 8 mm in diameter. Then investigated by a focused 1064 nm laser beam with pulse width τ_p = 10 ns and adjustable energy range (1−250 mJ). With the increasing of laser energy, the color of crystal surface changes. The superficial change of matrials were monitored by an optical microscope. Nova II sensor with a PE50-DIF-C energy sensor and a Vernier caliper was applied to measure the power of laser beam and the damage spot radius.

2. Computational Details

The electronic band structures, linear and nonlinear optical properties were performed using density functional theory (DFT) calculations by the *Vienna ab initio simulation package* (VASP)[6-8] with the Perdew-Burke-Ernzerhof (PBE)[9] exchange correlation functional. The projected-augmented plane-wave[10] pseudopotentials with the valence states Sr (4s, 4p and 5s), Cd (5s, 5p and 4d), Si (3s, 3p), S (3p) were used. In order to get convergent lattice parameters, a Γ-centered 5 × 5 × 5 Monkhorst-Pack grid for the Brillouin zone sampling[11] and a cutoff energy of 500 eV for the plane wave expansion were used. A Monkhorst-Pack *k*-point mesh of 9 × 9 × 9 was performed for linear and nonlinear optical calculation.

The imaginary part of the dielectric function due to direct inter-band transitions is given by the expression:

$$\varepsilon_2(\hbar\omega) = \frac{2e^2\pi}{\Omega\varepsilon_0}\sum_{k,v,c}\left|\langle\psi_k^c|u\cdot r|\psi_k^v\rangle\right|^2\delta(E_k^c - E_k^v - E) \quad \text{...........} (1)$$

where *Ω*, *ω*, *u*, *v* and *c* are the unit-cell volume, photon frequencies, the vector defining the polarization of the incident electric field, valence and conduction bands,

respectively. The real part of the dielectric function is obtained from ε_2 by a Kramers-Kronig transformation:

$$\varepsilon_1(\omega) = 1 + (\frac{2}{\pi})\int_0^{+\infty} d\omega' \frac{\omega'^2 \varepsilon_2(\omega)}{\omega'^2 - \omega^2} \quad \text{...........} \ (2)$$

The refractive index $n(\omega)$ can be obtained based on ε_1 and ε_2.

In calculation of the static $\chi^{(2)}$ coefficients, the so-called length-gauge formalism derived by Aversa and Sipe[10] and modified by Rashkeev et al[11] is adopted, which has proven to be successful in calculating the second order susceptibility for semiconductors and insulators. In the static case, the imaginary part of the static second-order optical susceptibility can be expressed as:

$$\chi^{abc} = \frac{e^3}{\hbar^2\Omega}\sum_{nml,k}\frac{r_{nm}^a(r_{ml}^b r_{ln}^c + r_{ml}^c r_{ln}^b)}{2\omega_{nm}\omega_{ml}\omega_{ln}}[\omega_n f_{ml} + \omega_m f_{ln} + \omega_l f_{nm}] + \frac{ie^3}{4\hbar^2\Omega}\sum_{nm,k}\frac{f_{nm}}{\omega_{mn}^2}[r_{nm}^a(r_{mn;c}^b + r_{mn;b}^c) + r_{nm}^b(r_{mn;c}^a + r_{mn;a}^c) + r_{nm}^c(r_{mn;b}^a + r_{mn;a}^b)]$$

...........(3)

where r is the position operator, $\hbar\omega_{nm} = \hbar\omega_n - \hbar\omega_m$ is the energy difference for the bands m and n, $f_{mn} = f_m - f_n$ is the difference of the Fermi distribution functions, subscripts a, b, and c are Cartesian indices, and $r^b_{mn;a}$ is the so-called generalized derivative of the coordinate operator in k space,

$$r_{nm;a}^b = \frac{r_{nm}^a\Delta_{mn}^b + r_{nm}^b\Delta_{mn}^a}{\omega_{nm}} + \frac{i}{\omega_{nm}} \times \sum_l(\omega_{lm}r_{nl}^a r_{lm}^b - \omega_{nl}r_{nl}^b r_{lm}^a) \quad \text{...........} \ (4)$$

where $\Delta^a_{nm} = (p^a_{nn} - p^a_{mm}) / m$ is the difference between the electronic velocities at the bands n and m.

As the nonlinear optical coefficients is sensitive to the momentum matrix, much finer k-point grid and large amount of empty bands are required to obtain a

convergent $\chi^{(2)}$ coefficient. The $\chi^{(2)}$ coefficients here were calculated from PBE wave functions and a scissor operator has been added to correct the conduction band energy (corrected to the experimental gap), which has proven to be reliable in predicting the second order susceptibility for semiconductors and insulators.

3. Figures and Tables

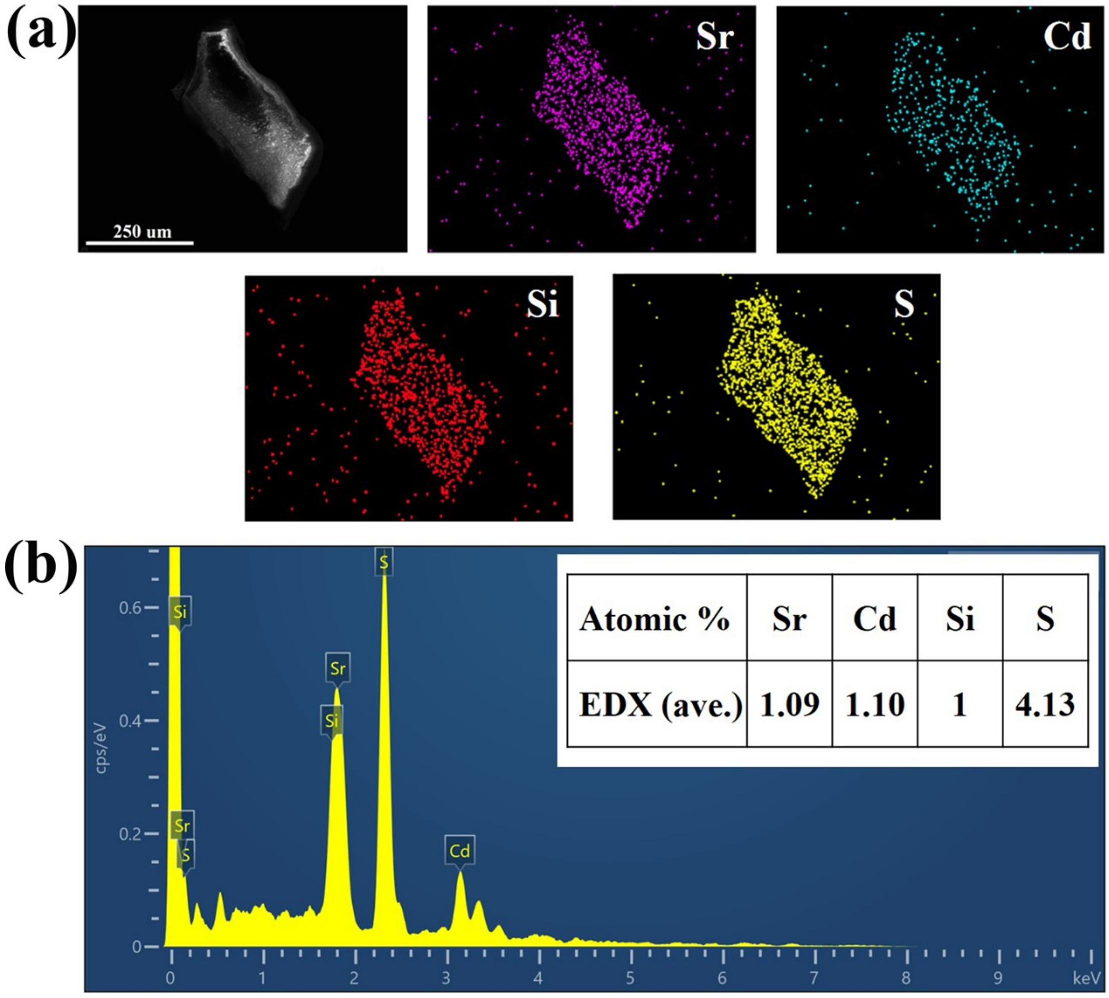

Figure S1. The characterizations of $SrCdSiS_4$: (a) SEM image and corresponding elemental mapping analysis and (b) EDX results.

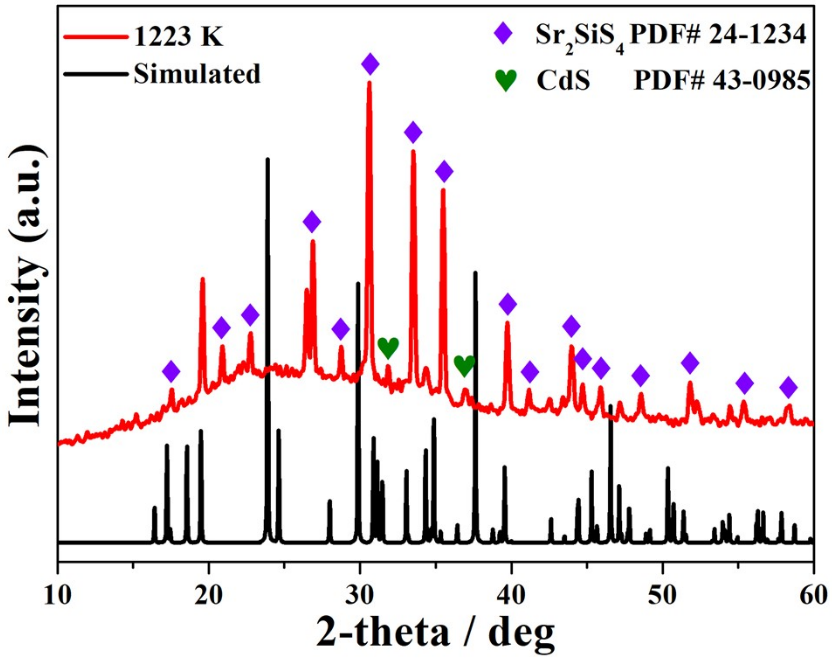

Figure S2. The experimental (red) and simulated (black) PXRD of $SrCdSiS_4$ calcining in 1223 K .

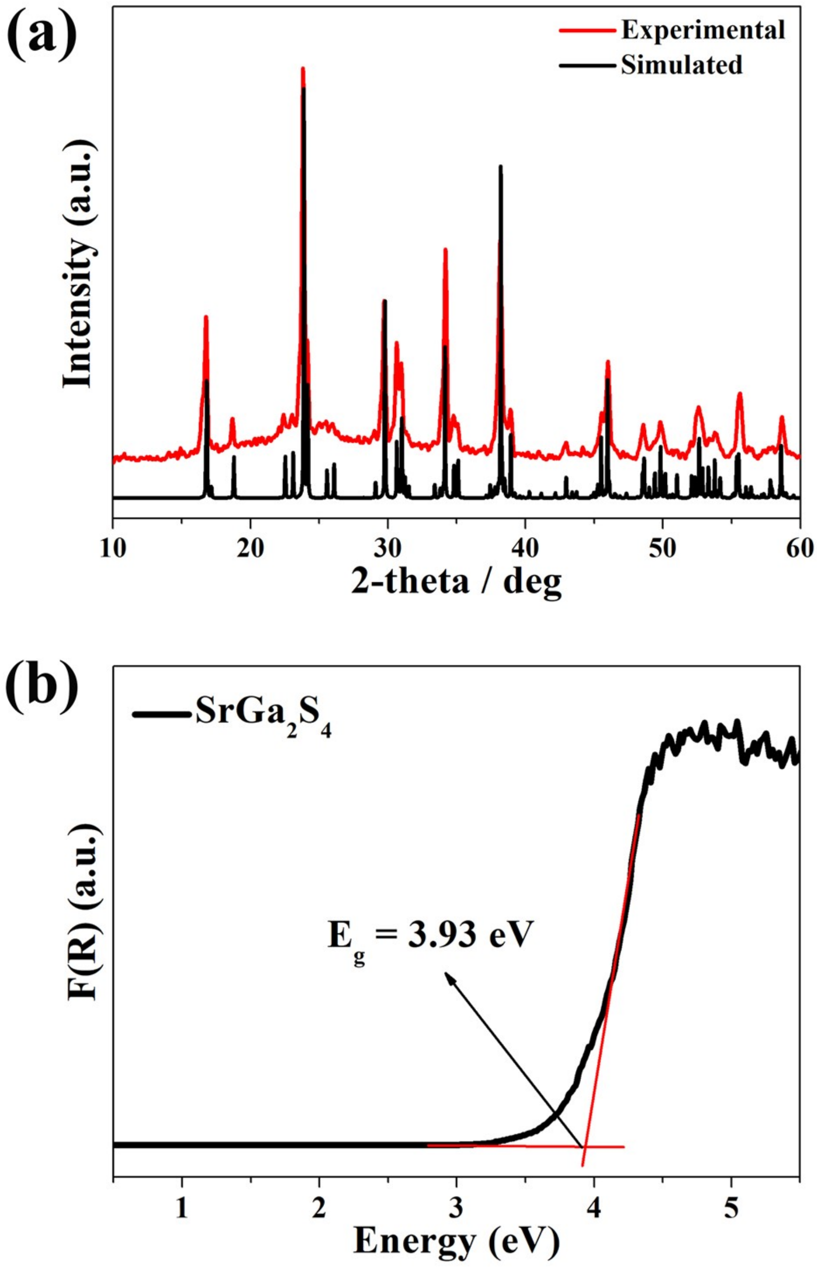

Figure S3. Property measurements for $SrGa_2S_4$: (a) the experimental (red) and simulated (black) PXRD; (b) solid-state diffuse reflectance spectrum.

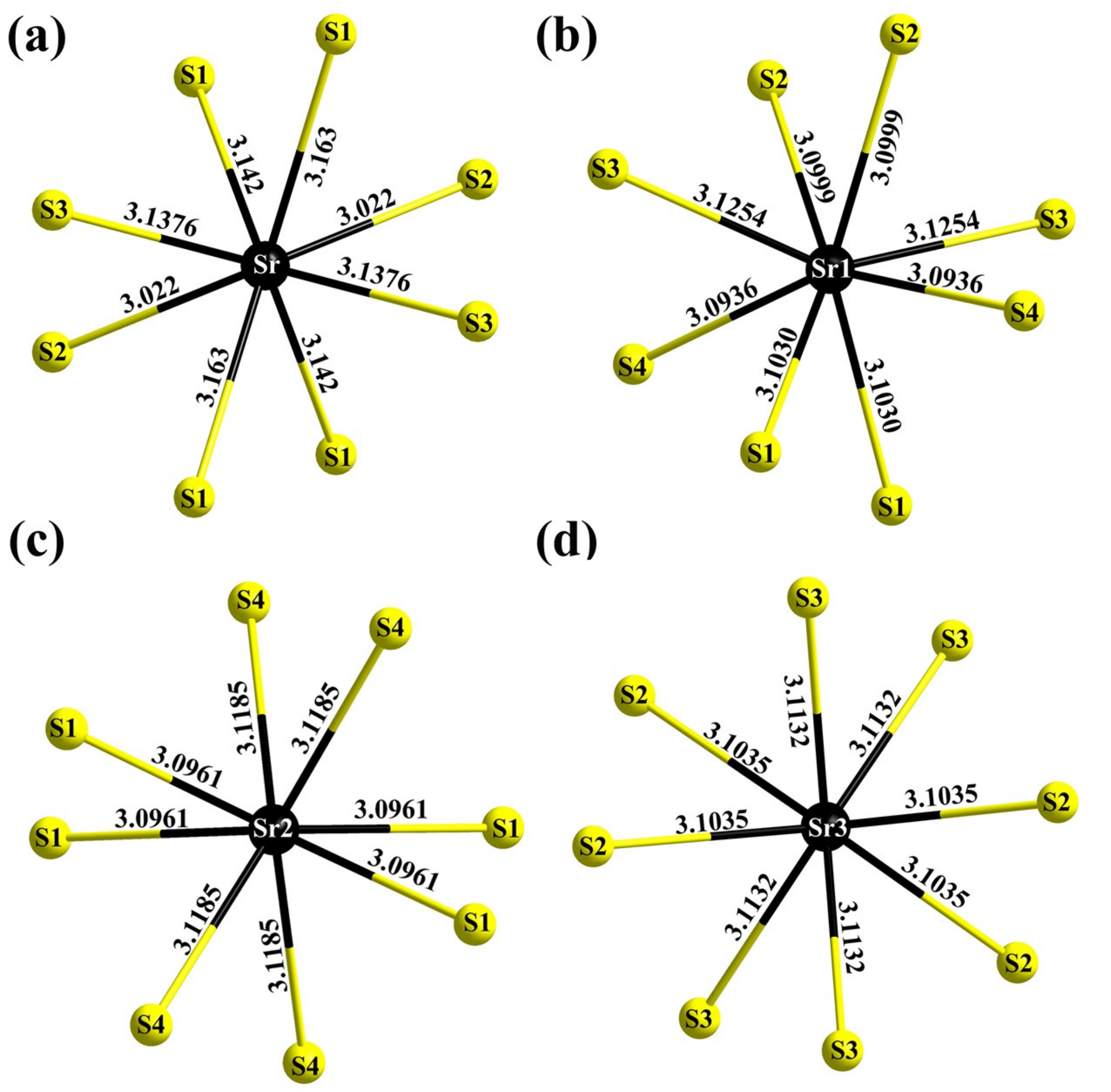

Figure S4. The coordination environment and bond lengths (Å) of crystallographic independent Sr atoms in (a) $SrCdSiS_4$ and (b–d) $SrGa_2S_4$.

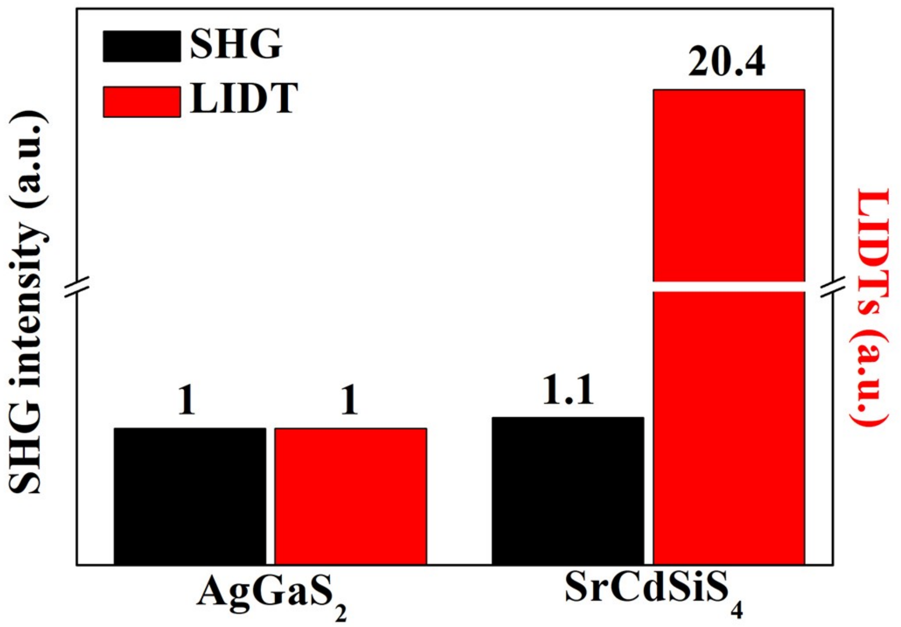

Figure S5. Comparison of the relative SHG and LIDT of $SrCdSiS_4$ and $AgGaS_2$ under the particle size of 150–210 μm.

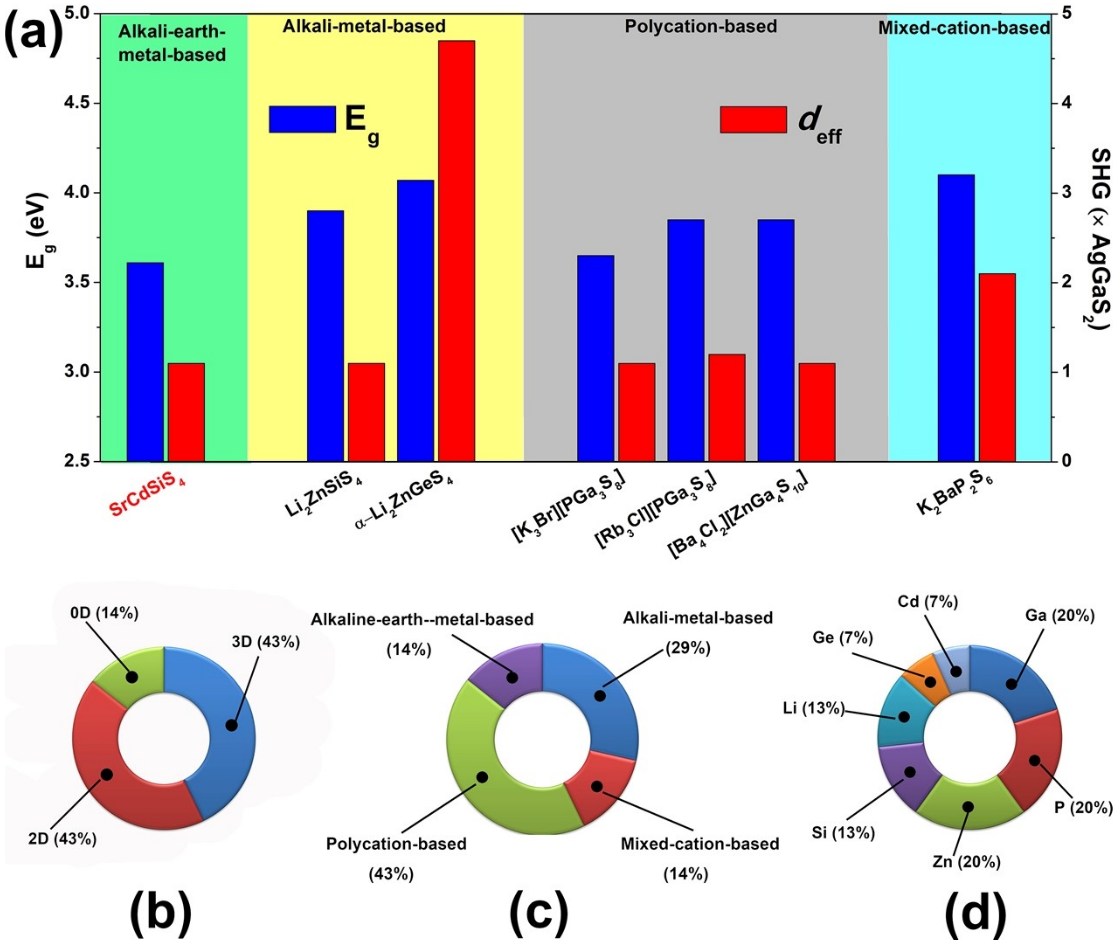

Figure S6. (a) Comparison of the reported PM IR-NLO chalcogenides with wide energy gaps (E_g > 3.5 eV) and strong SHG responses (d_{eff} > 1.0 × $AgGaS_2$). Distribution of 7 IR-NLO materials according to: (b) dimension, (c) type of filled cations, (d) central atom of BBUs.

Table S1 Property comparison of the known PM IR-NLO chalcogenides with wide band gaps (E_g > 3.5 eV) and strong SHG responses (d_{eff} > 1.0 × $AgGaS_2$).

	Compounds	Space group	Crystal system	E_g (eV)	d_{eff} (× $AgGaS_2$)	LIDT (× $AgGaS_2$)	Dimension	Ref.
Alkali-metal-based	Li_2ZnSiS_4	*Pna*2$_1$(no.33)	orthorhombic	3.9	1.1	10	3D	15
	α-Li_2ZnGeS_4	*Pmn*2$_1$(no.31)	orthorhombic	4.07	4.7	~40	3D	16
Polycation-baced	[Rb_3Cl][PGa_3S_8]	*Pmn*2$_1$(no.31)	orthorhombic	3.65	1.1	35	2D	17
	[K_3Br][PGa_3S_8]	*Pm*(no.6)	monoclinic	3.85	1.2	31	2D	17
	[Ba_4Cl_2][$ZnGa_4S_{10}$]	$I\bar{4}$(no.82)	tetragonal	3.85	1.1	51	3D	18
Mixed-cation based	$K_2BaP_2S_6$	*Pna*2$_1$(no.33)	orthorhombic	4.1	2.1	8	0D	19
Alkaline-earth-metal-based	**$SrCdSiS_4$**	***Ama*2(no.40)**	**orthorhombic**	**3.61**	**1.1**	**20.4**	**2D**	**This work**

Table S2 Selected bond lengths (Å) and angles (deg) of $SrCdSiS_4$.

Sr–S1×2	3.163(2)	∠S2–Cd–S1×2	135.67(6)
Sr–S1×2	3.142(2)	∠S2–Cd–S3	99.74(10)
Sr–S2×2	3.0220(16)	∠S1–Cd–S1	82.70(10)
Sr–S3×2	3.1376(18)	∠S1–Cd–S3×2	95.94(7)
Cd–S1×2	2.593(2)	∠S1–Si–S1	106.41(18)
Cd–S2	2.445(3)	∠S1–Si–S2×2	106.61(13)
Cd–S3	2.645(3)	∠S3–Si–S1×2	115.60(12)
Si–S1×2	2.139(3)	∠S3–Si–S2	105.33(19)
Si–S2	2.140 (4)		
Si–S3	2.107(4)		

Table S3 Selected bond lengths (Å) and angles (deg) of $SrGa_2S_4$.

Sr1–S1×2	3.1030(7)	∠S1–Ga1–S2	102.55(2)
Sr1–S2×2	3.0999(7)	∠S1–Ga1–S3	120.87(3)
Sr1–S3×2	3.1254(7)	∠S1–Ga1–S3	121.45(3)
Sr1–S4×2	3.0936(7)	∠S3–Ga1–S2	107.47(3)
Sr2–S1×4	3.0961(7)	∠S3–Ga1–S3	96.41(2)
Sr2–S4×4	3.1185(7)	∠S2–Ga2–S1	102.49(2)
Sr3–S2×4	3.1035(7)	∠S2–Ga2–S4	123.35(3)
Sr3–S3×4	3.1132(7)	∠S2–Ga2–S4	123.71(3)
Ga1–S1	2.2438(6)	∠S4–Ga2–S1	104.05(3)
Ga1–S2	2.3023(6)	∠S4–Ga2–S1	103.36(3)
Ga1–S3	2.2949(7)	∠S4–Ga2–S4	97.09(2)
Ga1–S3	2.3048(7)		
Ga2–S1	2.3016(6)		
Ga2–S2	2.2455(6)		
Ga2–S4	2.2976(7)		
Ga2–S4	2.2980(7)		

Table S4 Properties comparison of the reported $XM^{II}M^{IV}Q_4$ family (X = Eu, Sr, Ba; M^{II} = Mn, Zn, Cd, and Hg; M^{IV} = group-14 elements; and Q = chalcogen). The sequence of compounds is consistent with Figure 4.

Number	Compounds	Space group	E_g (eV)	SHG (×$AgGaS_2$)	LIDT (×$AgGaS_2$)	PM/NPM	Ref.
1	$SrZnGeS_4$	*Fdd*2 (no.43)	3.63	0.9@150–212	35.0	PM	20
2	$BaZnSnS_4$	*Fdd*2 (no.43)	3.25	0.6@210–250	9.8	PM	21
3	$SrZnSnS_4$	*Fdd*2 (no.43)	2.83	0.4@80–100	N/A	PM	22
4	β-$BaHgSnS_4$	*Ama*2 (no.40)	2.77	2.8@150–200	N/A	PM	23
5	$SrHgSnS_4$	*Ama*2 (no.40)	2.72	1.9@150–200	N/A	PM	23
6	$BaZnSiSe_4$	*Ama*2 (no.40)	2.71	0.3@41–74	N/A	N/A	24
7	$SrCdGeS_4$	*Ama*2 (no.40)	2.6	1.7@150–200	N/A	PM	25
8	$BaCdGeS_4$	*Fdd*2 (no.43)	2.58	0.3@200–250	13.0	PM	26
9	$EuCdGeS_4$	*Ama*2 (no.40)	2.5	2.6@150–200	N/A	PM	27
10	$BaHgGeSe_4$	*Ama*2 (no.40)	2.49	4.7@150–200	N/A	PM	28
11	$BaZnGeSe_4$	*Ama*2 (no.40)	2.46	1.0@41–74	N/A	PM	24
12	$SrHgGeSe_4$	*Ama*2 (no.40)	2.42	4.8@150–200	N/A	PM	28

13	$BaCdSnS_4$	*Fdd*2 (no.43)	2.30	5.0@38–55	N/A	NPM	29
14	$EuCdGeSe_4$	*Ama*2 (no.40)	2.25	3.8@150–200	N/A	PM	27
15	$SrZnSnSe_4$	*Fdd*2 (no.43)	2.14	0.3@20–41	5.0	NPM	30
16	$EuHgSnS_4$	*Ama*2 (no.40)	2.14	1.8@150–200	N/A	PM	31
17	$SrHgSnSe_4$	*Fdd*2(no.43)	2.07	4.9@150–200	N/A	PM	23
18	$SrCdSnS_4$	*Fdd*2 (no.43)	2.05	1.3@200–250	10.0	PM	32
19	$EuHgGeS_4$	*Ama*2 (no.40)	2.04	0.9@150–210	N/A	PM	33
20	$BaHgSnSe_4$	*Fdd*2 (no.43)	1.98	5.1@150-200	N/A	PM	23
21	$EuHgGeSe_4$	*Ama*2 (no.40)	1.97	3.1@150-200	N/A	PM	31
22	$BaMnSnS_4$	*Fdd*2 (no.43)	1.9	1.2@200-250	10	PM	26
23	$SrCdGeSe_4$	*Ama*2 (no.40)	1.9	5.3@150-200	N/A	PM	25
24	$BaZnSnSe_4$	*Fdd*2 (no.43)	1.88	1@210-250	5.7	PM	21
25	$SrZnSnSe_4$	*Fdd*2 (no.43)	1.82	5@70-90	N/A	PM	34
26	$BaCdSnSe_4$	*Fdd*2 (no.43)	1.79	1.6@38-55	N/A	NPM	35
27	$BaCdGeSe_4$	*Fdd*2 (no.43)	1.77	0.5@150-210	N/A	PM	36
28	$SrCdSnSe_4$	*Fdd*2 (no.43)	1.54	1.5@200-250	5.0	PM	32

*****	**$SrCdSiS_4$**	***Ama*2 (no.40)**	**3.61**	**1.1@150-210**	**20.4**	**PM**	**This work**

4. References

1. J. Tauc, R. Grigorovici and A. Vancu, *Phys. Status Solidi B,* 1966, **15**, 627–637.
2. CrystalClear Version 1.3.5; Rigaku Crop.: Woodlands, TX, 1999.
3. P. Kubelka, *Z. Tech. Phys.,* 1931, **12**, 593–601.
4. S. K. Kurtz and T. T. Perry, *J. Appl. Phys.,* 1968, **39**, 3798–3813.
5. M.-J. Zhang, X. M. Jiang, L. J. Zhou and G. C. Guo, *J. Mater. Chem. C,* 2013, ***1*,** 4754–4760.
6. G. Kresse and J. Furthmuller, *Phys. Rev. B: Condens. Matter,* 1996, **54**, 11169–11186.
7. G. Kresse and D. Joubert, *Phys. Rev. B: Condens. Matter,* 1999, **59,** 1758–1775.
8. G. Kresse, VASP, 5.3.5; http://cms.mpi.univie.ac.at/vasp/vasp/vasp.html.
9. P. E. Blochl, *Phys. Rev. B: Condens. Matter,* 1994, **50,** 17953–17979.
10. J. P. Perdew, K. Burke and M. Ernzerhof, *Phys. Rev. Lett.,* 1996, **77**, 3865–3868.
11. H. J. Monkhorst and J. D. Pack, *Phys. Rev. B: Condens. Matter,* 1976, **13**, 5188–5192.
12. J. A. Brant, D. J. Clark, Y. S. Kim and J. I. Jang, *Inorg. Chem.,* 2015, **54**, 2809–2819.
13. J. A. Brant, D. J. Clark, Y. S. Kim, J. I. Jang, J.-H. Zhang and J. A. Aitken, *Chem. Mater.,* 2014, **26,** 3045–3048.
14. M. M. Chen, S. H. Zhou, W. B. Wei, X. T. Wu, H. Lin and Q. L. Zhu, *Adv. Opt. Mater.,* 2022, **10,** 2102123.
15. G. Li, Y. Chu and Z. Zhou, *Chem. Mater.,* 2018, **30,** 602–606.
16. J.-H. Zhang, D. J. Clark, J. A. Brant, K. A. Rosmus, P. Grima, J. W. Lekse, J. I. Jang and J. A. Aitken, *Chem. Mater.,* 2020, **32,** 8947–8955.
17. B. W. Liu, H. Y. Zeng, X. M. Jiang, G. E. Wang, S. F. Li, L. Xu and G. C. Guo,

Chem. Sci., 2016, **7,** 6273–6277.

18. H. Chen, Y.-Y. Li, B. Li, P.-F. Liu, H. Lin, Q.-L. Zhu and X.-T. Wu, *Chem. Mater.,* 2020, **32,** 8012–8019.
19. V. Nguyen, B. Ji, K. Wu, B. Zhang and J. Wang, *Chem. Sci.,* 2022, **13,** 2640–2648.
20. Q. Q. Liu, X. Liu, L. M. Wu and L. Chen, *Angew. Chem. Int. Ed.,* 2022, DOI: 10.1002/anie.202205587.
21. Y.-N. Li, Z.-X. Chen, W.-D. Yao, R.-L. Tang and S.-P. Guo, *J. Mater. Chem. C,* 2021, ***9,*** 8659–8665.
22. Y. Zhang, D. Mei, Y. Yang, W. Cao, Y. Wu, J. Lu and Z. Lin, *J. Mater. Chem. C,* 2019, **7,** 8556–8561.
23. Y. Guo, F. Liang, Z. Li, W. Xing, Z. S. Lin, J. Yao, A. Mar and Y. Wu, *Inorg. Chem.,* 2019, **58**, 10390–10398.
24. W. Yin, A. K. Iyer, C. Li, J. Yao and A. Mar, *J. Alloys Compd.,* 2017, **708**, 414–421.
25. Y. Dou, Y. Chen, Z. Li, A. K. Iyer, B. Kang, W. Yin, J. Yao and A. Mar, *Cryst. Growth Des.,* 2019, **19**, 1206–1214.
26. Y.-J. Lin, R. Ye, L.-Q. Yang, X.-M. Jiang, B.-W. Liu, H.-Y. Zeng and G.-C. Guo, *Inorg. Chem. Front.,* 2019, **6**, 2365–2368.
27. W. Xing, N. Wang, Y. Guo, Z. Li, J. Tang, K. Kang, W. Yin, Z. Lin, J. Yao and B. Kang, *Dalton Trans.,* 2019, **48**, 17620–17625.
28. Y. Guo, F. Liang, W. Yin, Z. Li, X. Luo, Z.-S. Lin, J. Yao, A. Mar and Y. Wu, *Chem. Mater.,* 2019, **31**, 3034–3040;
29. N. Zhen, K. Wu, Y. Wang, Q. Li, W. Gao, D. Hou, Z. Yang, H. Jiang, Y. Dong and S. Pan, *Dalton Trans.,* 2016, **45**, 10681–10688.

30. X. Pang, R. Wang, X. Che and F. Huang, *J. Solid State Chem.,* 2021, **297**, 122092.

31. W. Xing, C. Tang, N. Wang, C. Li, Z. Li, J. Wu, Z. Lin, J. Yao, W. Yin and B. Kang, *Inorg. Chem.,* **2020**, **59**, 18452–18460.

32. Y.-J. Lin, B.-W. Liu, R. Ye, X.-M. Jiang, L.-Q. Yang, H.-Y. Zeng and G.-C. Guo, *J. Mater. Chem. C,* 2019, **7,** 4459–4465;

33. M. Yan, Z.-D. Sun, W.-D. Yao, W. Zhou, W. Liu and S.-P. Guo, *Inorg. Chem. Front.,* 2020, **7,** 2451–2458.

34. F. Hou, D. Mei, Y. Zhang, F. Liang, J. Wang, J. Lu, Z. Lin and Y. Wu, *J. Alloys Compd.,* 2022, **904,** 163944.

35. K. Wu, X. Su, Z. Yang and S. Pan, *Dalton Trans.,* 2015, **44**, 19856–19864.

36. F.-Y. Yuan, C.-S. Lin, Y.-Z. Huang, H. Zhang, A.-Y. Zhou, G.-L. Chai and W.-D. Cheng, *J. Solid State Chem.,* 2021, **302**, 122352.
